# Identifying Persuasive Design Principles and Behavior Change Techniques Supporting End User Values and Needs in eHealth Interventions for Long-Term Weight Loss Maintenance: Qualitative Study

**DOI:** 10.2196/22598

**Published:** 2020-11-30

**Authors:** Rikke Aune Asbjørnsen, Jobke Wentzel, Mirjam Lien Smedsrød, Jøran Hjelmesæth, Matthew M Clark, Lise Solberg Nes, Julia E W C Van Gemert-Pijnen

**Affiliations:** 1 Centre for eHealth and Wellbeing Research Department of Psychology, Health and Technology University of Twente Enschede Netherlands; 2 Research and Innovation Department Vestfold Hospital Trust Tønsberg Norway; 3 Department of Digital Health Research Division of Medicine Oslo University Hospital Oslo Norway; 4 Research Group IT Innovations in Health Care Windesheim University of Applied Sciences Zwolle Netherlands; 5 Collaborative Care Unit Sørlandet Hospital Trust Kristiansand Norway; 6 Morbid Obesity Center Vestfold Hospital Trust Tønsberg Norway; 7 Department of Endocrinology, Morbid Obesity and Preventive Medicine Institute of Clinical Medicine University of Oslo Oslo Norway; 8 Department of Psychiatry & Psychology Mayo Clinic College of Medicine & Science Rochester, MN United States; 9 Institute of Clinical Medicine Faculty of Medicine University of Oslo Oslo Norway; 10 University Medical Center Groningen Groningen Netherlands; 11 University of Waterloo Waterloo, ON Canada

**Keywords:** eHealth, weight loss maintenance, behavior change, design thinking, digital health interventions, persuasive technology, human-centered design

## Abstract

**Background:**

An increasing number of eHealth interventions aim to support healthy behaviors that facilitate weight loss. However, there is limited evidence of the effectiveness of the interventions and little focus on weight loss maintenance. Knowledge about end user values and needs is essential to create meaningful and effective eHealth interventions, and to identify persuasive system design (PSD) principles and behavior change techniques (BCTs) that may contribute to the behavior change required for successful long-term weight loss maintenance.

**Objective:**

This study aimed to provide insight into the design of eHealth interventions supporting behavior change for long-term weight maintenance. The study sought to identify the values and needs of people with obesity aiming to maintain weight after weight loss, and to identify PSD principles, BCTs, and design requirements that potentially enable an eHealth intervention to meet end user values and needs.

**Methods:**

This study presents the concept of integrating PSD principles and BCTs into the design process of eHealth interventions to meet user values and needs. In this study, individual interviews and focus groups were conducted with people with obesity (n=23) and other key stakeholders (n=27) to explore end user values and needs related to weight loss maintenance. Design thinking methods were applied during the focus group sessions to identify design elements and to explore how eHealth solutions can support the needs to achieve sustainable weight loss maintenance. The PSD model and behavior change taxonomy by Michie were used to identify PSD principles and BCT clusters to meet end user values and needs.

**Results:**

A total of 8 key end user values were identified, reflecting user needs for weight loss maintenance support: *self-management*, *personalized care*, *autonomy*, *feel supported*, *positive self-image*, *motivation*, *happiness*, and *health*. *Goals and planning*, *feedback and monitoring*, *repetition and substitution*, *shaping knowledge*, *social support*, *identity*, and *self-belief* were some of the BCT clusters identified to address these concepts, together with PSD principles such as *personalization*, *tailoring*, *self-monitoring*, *praise*, and *suggestions*.

**Conclusions:**

The process of translating end user values and needs into design elements or features of eHealth technologies is an important part of the design process. To our knowledge, this is the first study to explore how PSD principles and BCTs can be integrated when designing eHealth self-management interventions for long-term weight loss maintenance. End users and other key stakeholders highlighted important factors to be considered in the design of eHealth interventions supporting sustained behavior change. The PSD principles and BCTs identified provide insights and suggestions about design elements and features to include for supporting weight loss maintenance. The findings indicate that a combination of BCTs and PSD principles may be needed in evidence-based eHealth interventions to stimulate motivation and adherence to support healthy behaviors and sustained weight loss maintenance.

**Trial Registration:**

ClinicalTrials.gov NCT04537988; https://clinicaltrials.gov/ct2/show/NCT04537988

## Introduction

### The Challenges of Weight Loss Maintenance

Globally, obesity has grown to epidemic proportions and nearly tripled between 1975 and 2016 [1]. In 2016, more than 650 million adults worldwide were obese [1], which led to individual and societal challenges with regard to health, well-being, and economic burden [2-5]. Many people with obesity manage to lose weight, but an alarmingly large percentage of people (3 out of 4) fail to maintain the lost weight over time [6]; therefore, long-term weight maintenance following weight loss is a major concern.

Sustained health behavior change is required to prevent weight regain [7-9], and the chance of success increases when individuals maintain healthy behaviors and weight loss for 2 to 5 years [10,11]. The US National Weight Control Registry [12] and several weight maintenance–related studies provide information about factors [13] associated with successful long-term weight maintenance [8-12]. The identified factors include continued adherence to behavioral strategies such as frequent self-weighing and habitual routines, including high levels of physical activity, healthy diet, eating breakfast regularly, and consistent eating patterns across weekdays and weekends [8,10,13-20].

The challenges that people face when trying to prevent weight regain after weight loss are complex, with several biological, behavioral, cognitive, emotional, social, and environmental factors interacting [16,21]. To address these challenges, novel solutions and emerging technologies, such as eHealth solutions, have the potential to support the self-management and behavior change processes needed for continued weight control [17,21-26]. At present, several eHealth interventions are available to support weight loss [23,27-29]. However, evidence of long-term effects related to weight loss maintenance solutions is limited. Research examining the potential of design for sustained behavior change through eHealth interventions [27] focusing on weight loss maintenance is therefore needed [30,31].

### Combining Persuasive and Behavior Change Techniques in eHealth Design

eHealth interventions and persuasive technologies offer possibilities for improving self-management of health and are increasingly used to support healthy behaviors for improved health and well-being [26,28-32]. Establishing habits for long-term behavior change and weight loss maintenance is challenging and takes time [10,33]. For long-term behavior change, eHealth interventions need to provide effective self-management strategies, support lifestyle change, and promote healthy behaviors. A major challenge to effective eHealth is to integrate motivating and engaging design elements for adherence and continuity of use [23,34,35]. Despite these challenges, such technologies have the potential to empower individuals and transform health care by shifting the focus from cure to prevention and self-management with improved health outcomes [36-38]. This requires not only attention to existing evidence from research and clinical practice but also careful design in collaboration with end users and other stakeholders to develop and implement feasible and sustainable eHealth solutions [36,39-42]. In the design of eHealth interventions, behavior change techniques (BCTs) [43] and persuasive system design (PSD) principles [44] are increasingly applied to design effective behavioral interventions that motivate, engage, and promote healthy lifestyles in support of continued behavior change [45-47]. Behavior change taxonomy by Michie [43] has been systematically developed to meet the need for standardized reporting when designing and evaluating complex behavior change interventions, building on a broad range of BCTs to describe intervention components and content, independent of theory. The PSD model by Oinas-Kukkonen [26] includes a range of PSD principles that can be applied when designing novel and user-friendly eHealth interventions and persuasive technology to support healthy behaviors and behavior change. However, the most effective combination of such techniques and principles remains unclear [23,26,36,43,47-55].

The research team of this study had recently conducted a scoping review [23] that identified PSD principles [26] (eg, *personalization*, *self-monitoring*, *tailoring*, *praise*, *suggestions*, *rewards*, and *reminders*) and BCT clusters [43] (eg, *feedback and monitoring*, *goals and planning*, *social support*, *shaping knowledge*, *associations*, and *repetition* and *substitution*) applied in eHealth interventions to stimulate motivation, adherence, and weight loss maintenance [23]. Findings from the review suggest different strategies for losing weight than for weight maintenance [23]. Although eHealth interventions, when developed in line with user needs [36,56,57], have the potential to support the difficult behavior change process needed to prevent long-term weight regain [10,21,23,58-60], the scoping review revealed that user involvement in the design and development of such eHealth interventions is lacking [23]. Moreover, there is insufficient knowledge about the most ideal combinations of PSD principles and BCTs to support weight maintenance over time [23]. To create meaningful and effective digital behavior change interventions, it is essential to identify the end user values and needs and the related PSD principles and BCTs during the requirement specification process [23,32,36,39,61]. A holistic and broad stakeholder-driven approach, including key stakeholders, is imperative to design and develop sustainable eHealth interventions that reflect the values and support the goals of the end users [36,39].

### Objectives

The overall aim of this study was to provide insight into the design of eHealth interventions aiming to support behavior change for long-term weight loss maintenance. The goal was to identify values and needs of people with obesity aiming to maintain weight after weight loss (ie, end users) and how these values and needs can be met by PSD principles and BCTs in eHealth intervention design. The following research questions were addressed:

1. What are the values and needs of people with obesity (ie, end users) aiming to maintain weight after initial weight loss, according to key stakeholders (eg, end users and health care providers)?
2. Which PSD principles, BCTs, and design requirements can be identified to meet end user values and needs?

End user *values* refer to the main drivers of behavior or high-level needs or requirements (eg, improved health and motivation to maintain healthy routines in the long term), indicating the added values of the technology or a solution to support users in reaching their goals (ie, maintaining weight after weight loss) [36,39]. End user *needs* refer to demands and low-level requirements (ie, low-level needs) that people want to solve to address their problems (eg, realistic goal setting, self-regulation, and coping skills to maintain weight) [36,39]. A value can drive several underlying needs [36,39,62].

A *key stakeholder* was defined as someone central in understanding end user challenges and needs, including possible latent and future needs, and someone that could provide valuable input on how to meet end user needs in eHealth technology design [36,63].

## Methods

### The Double Diamond Framework and the Center for eHealth Research and Disease Management Roadmap

**Figure 1:**
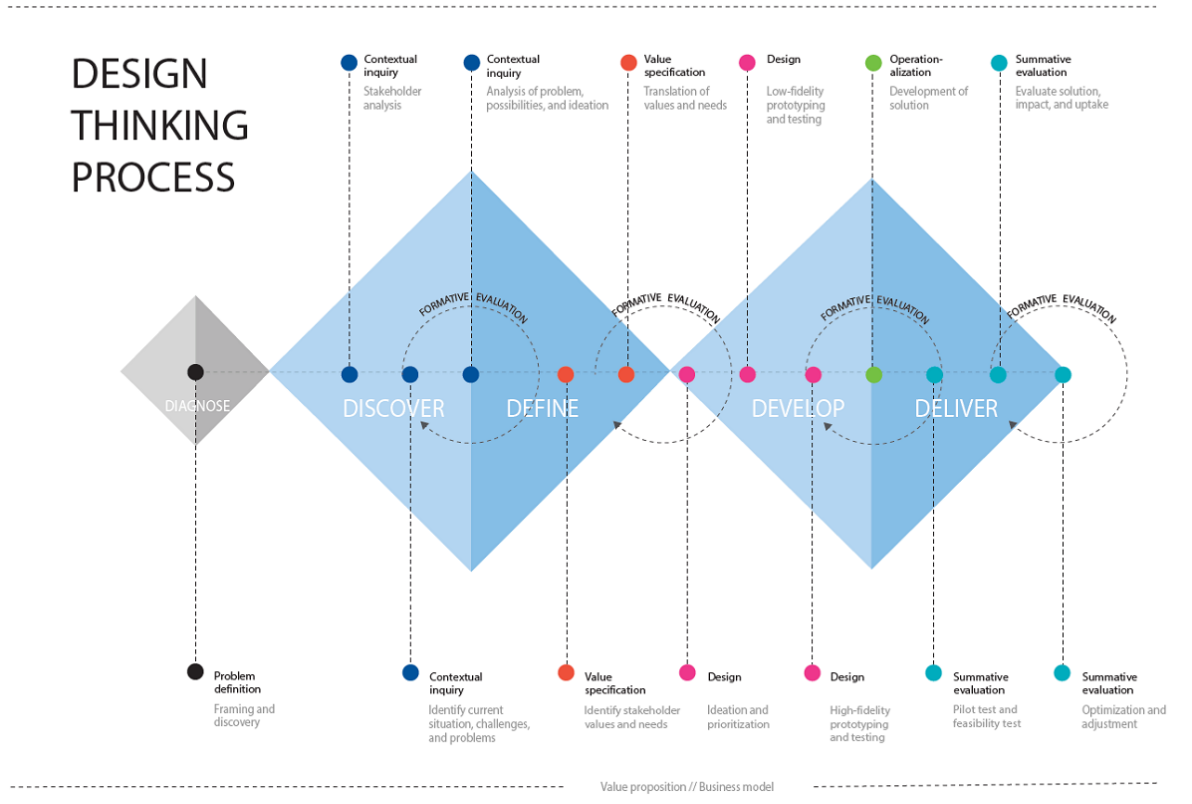
The Double Diamond [64] and the Center for eHealth Research and Disease Management roadmap [39] combined: a design thinking process for eHealth design and development.

This study builds on the innovation and design framework of the Double Diamond [64,65] and the Center for eHealth Research and Disease Management (CeHRes) comprehensive roadmap [36,39] to guide the design and development of an evidence-based eHealth intervention supporting long-term behavior change and weight loss maintenance. Figure 1 presents a combination of the Double Diamond [64] and the CeHRes roadmap [36,39].

As a first step, a *diagnose phase* was included [66,67], consisting of the previously performed scoping review [23] and an alignment workshop with the research group (including a user representative) to define the issues at hand (ie, problem definition) and create an initial overview of the stakeholders in the weight loss maintenance conundrum.

The shape of the Double Diamond represents the process of gathering insights (diverging to discover) and translating these insights into ideas or concepts (converging to define) [21,64,65,68-72]. The Double Diamond framework utilizes design thinking methods that can be applied to identify user needs and to find innovative solutions for complex health care challenges [63,70,71,73]. Design thinking explores and elaborates on what is desirable from a user perspective, what is technologically feasible, and what is economically viable [74]. Complementing the design thinking process, the CeHRes roadmap [36,39] was applied as a guideline for holistic development, implementation, and evaluation of eHealth technologies. The CeHRes roadmap consists of the following iterative phases: *contextual inquiry*, *value specification*, *design*, *operationalization*, and *summative evaluation* [36,39]. The roadmap integrates concepts of persuasive technology design [26], human-centered design, and business modeling through participatory development with continuous evaluation cycles during the eHealth development and implementation process [39].

This study focuses on the *discover* and *define* phase [64] (Figure 1), including the *contextual inquiry* and *value specification* phases [39]. During the *discover* phase [64], a stakeholder analysis [63] was executed to identify key stakeholders who could provide insights into the needs, challenges and problems of people aiming to maintain weight after weight loss (ie, end users) [63]. As part of the *contextual inquiry*, individual interviews and focus group sessions were performed to elicit end user values and needs and to inquire how needs can be met by eHealth technology [36]. During the *define* phase [64], the values and needs of end users were identified, resulting in *value specification* [39]. Finally, the PSD model [26], behavior change taxonomy by Michie [43], and the results from the recent scoping review [23] were used to translate values and needs into PSD principles and BCTs [39,75,76].

### Key Stakeholder Identification and Recruitment

#### Stakeholder Analysis

**Figure 2:**
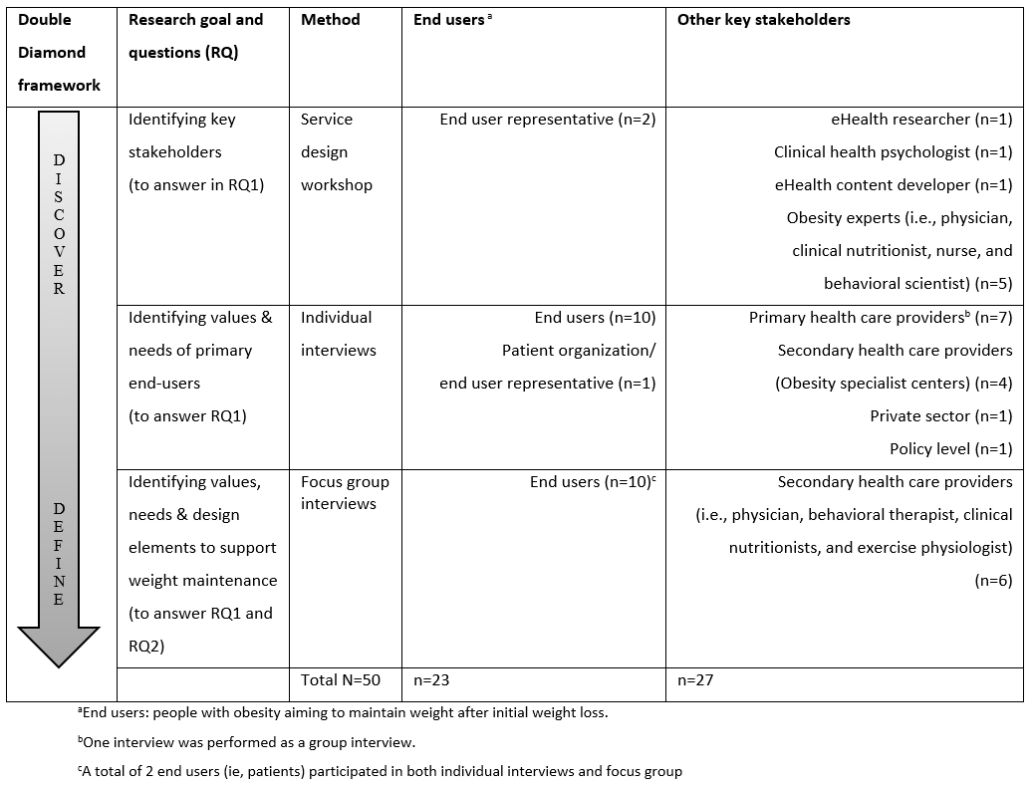
Study participants and data collection.

To identify key stakeholders, as part of the *discover* phase, a service design workshop [63] was organized with a multidisciplinary team of stakeholders, consisting of end user representatives (ie, people with obesity aiming to maintain weight after initial weight loss; n=2), health care personnel (ie, health care providers; n=5), and project group representatives (n=3). See Figure 2 for study participants, including the participants in the service design workshop.

The stakeholder identification workshop was led by a service designer and aimed to create a map of key stakeholders [63] to involve in the research and design process. On the basis of the methods from stakeholder theory and service design [36,63,73,77,78], idea generation was facilitated during the workshop by using a large whiteboard stakeholder map as a mind mapping tool [63], generating a preliminary list of potential stakeholders. The initial list of stakeholders was then narrowed to key stakeholders based on group discussions and mutual agreement during the workshop session. The broad and multidisciplinary stakeholder involvement in this study is part of a holistic and human-centered development [36,39,70,73].

#### Recruitment of End Users and Other Key Stakeholders

Prospective *end users* (ie, people with obesity aiming to maintain weight after initial weight loss—the intervention target group) were defined as adults aged 18 years or above, with BMI ≥30 kg/m^2^ [79] before weight loss, who had recently lost 8% weight or more (eg, through a low-calorie diet or lifestyle change program), and who were in need of support to prevent weight regain. People who met these criteria and had previously experienced weight loss, which was followed by weight regain and/or weight maintenance, were selected for study participation. Eligible participants also had to have access to the internet and be fluent in the Norwegian language. Recruitment was conducted over several weeks by 3 collaborative leading obesity research and treatment centers in Norway through convenience sampling, that is, people meeting the inclusion criteria were invited to participate in the study by their health care provider when undergoing treatment or during outpatient follow-up visits. Potential study participants (ie, prospective end users) received written and oral study information, and were invited to participate in individual interviews and focus groups at local hospitals and research centers (Figure 2). If interested, the potential participants were contacted by the research team, provided with an appointment time, and informed that they would receive a gift certificate (approximate value US $30) as compensation for time spent or of potential costs (eg, transport and parking). Representatives of *other key stakeholders* identified during the stakeholder analysis (eg, health care providers) were contacted based on convenience sampling and invited to participate in individual interviews and focus groups through the collaborating health care institutions. See Figure 2 for details.

#### Ethical Approval and Informed Consent

This study was approved by the Hospital Privacy and Security Protection Committee (institutional review board, approval number: 2017/12702) at Oslo University Hospital (OUH) in Norway, one of the largest medical centers in northern Europe. All participants involved in this study received study information and signed a written informed consent form before participation.

### Identification of End User Values and Needs

#### Semistructured Interviews

As part of the data collection, individual semistructured interviews were performed with prospective end users (n=11) who had recently lost weight and needed support and guidance to prevent weight regain. Other key stakeholders (n=13) were also interviewed to capture a broad perspective and understanding of end user challenges, values, and needs to maintain weight after weight loss [36,73,80] (Figure 2). Interviews with end users and other key stakeholders were guided by the overarching research question: *What are the values and needs of people with obesity aiming to maintain weight after initial weight loss?*

Individual interviews (60-min to 90-min long) were performed and audio recorded by the first and third author (RA: 17/23, 73%; MS: 6/23, 26%). A semistructured interview guide was developed (Multimedia Appendix 1), providing an overview of the themes to explore. These themes included everyday life, behavior, thoughts, feelings, routines, challenges, strategies, values and needs to maintain weight, experiences and preferences related to health apps, activity and nutrition tracker, and weight management technology (eg, apps, health forums, web-based weight management programs, videos, blogs, and wearables).

#### Focus Groups

To further explore and validate the everyday needs of end users and elaborate on engaging and motivating design elements that could potentially support weight maintenance in eHealth design, 3 focus group sessions were held [80-82]. In 2 focus groups, 10 end users (n=10) participated, 5 in each group. In the third focus group, other key stakeholders (ie, health care personnel) participated (n=6) [81,82] (Figure 2). The focus groups lasted approximately 2.5 hours and were facilitated by a service designer and the first author (RA). A digital designer and a Scrum (ie, Agile project management methodology or framework) product owner also participated in the focus groups. The focus group sessions had an explorative and creative character, applying participatory and ideation methods from design thinking [73]. All 3 focus groups were given the following assignment and topics to discuss.

First, a “What is your favorite app?” assignment was performed, where participants described and/or suggested their own apps. The assignment was performed to stimulate creativity and to create trust and social connections within the group. During the assignment, positive and negative aspects of the apps were elaborated on employing brainstorming techniques. The following topics were then explored to provide input for the main research questions:

- What do you/end users need to maintain weight?
- Which design elements can help you/end users to maintain weight?

Sticky notes and a large whiteboard were used to discuss these topics (ie, open-ended questions) with the focus group participants, eliciting potential unmet user needs. This technique was used to create an image of the diversity of user needs [73]. A dynamic and informal discussion, where focus group participants could instantly react to each other’s suggestions, provided the researchers with a broad impression of the topics [81].

To identify engaging and motivating design elements, feature cards were applied based on design methods and principles from open card sorting (although applied in a less traditional way than card sorting tasks for information architecture; Multimedia Appendix 2) [83-87]. The cards contained PSD principles and BCTs identified in the previously mentioned scoping review [23]. Additional design elements were included to explore other potentially engaging elements supporting behavior change and to facilitate the creative process related to users’ requirements, preferences, and ideas to meet their needs [23,86-90]. These elements include metaphors (eg, when a target is reached, a flower grows in a garden), avatars (eg, a virtual or digital person based on oneself), and gamification elements (eg, points and trophies).

The participants could also choose to develop their own cards with motivational elements through sketching or writing, as some cards had no content (ie, were blank). Examples of how the design elements could be used in app design were presented during the introduction. The focus group participants could choose and sort the cards they identified as useful (ie, to motivate and support weight maintenance) and then share their results in an open group discussion. Participants sorted the selected cards into groups of *must have*, *nice to have*, and *not needed*.

### Translation of Values and Needs Into PSD Principles, BCTs, and Design Requirements

**Figure 3:**
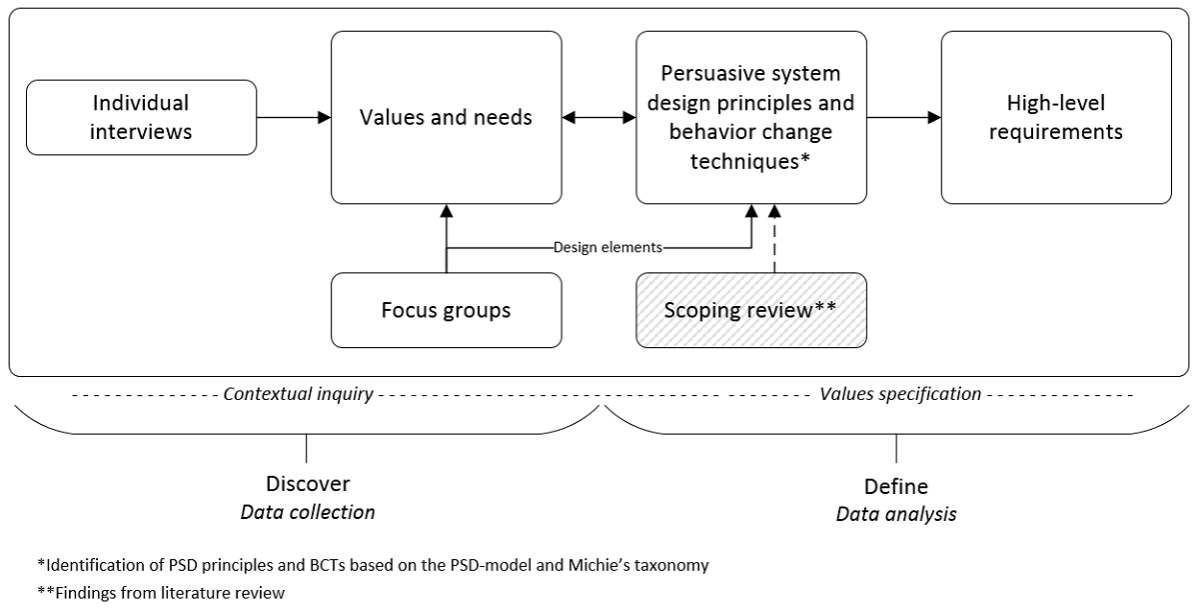
Value identification and translation of identified values and needs into persuasive system designs, behavior change techniques, and high-level requirements. BCT: behavior change technique; PSD: persuasive system design.

The requirement elicitation methods (ie, individual interviews, focus groups) aimed to capture the user perception of what end users need and how the design of an eHealth technology should be [36]. A total of 3 researchers (RA, MS, and JW) participated in the analysis and validation process of values and needs before translating these into PSD principles, BCTs, and high-level requirements for the design and development of an eHealth weight loss maintenance intervention [91] (Multimedia Appendix 3) [76]. Figure 3 illustrates the steps used when translating end user values and needs into PSD principles, BCT clusters, and high-level design requirements. The presented methods provided input for the main research question: *Which PSD principles, BCTs, and design requirements can be identified to meet the end user´ values and needs?*

### Data Analysis

#### Semistructured Interviews

As part of the *define* phase, verbatim transcription of audio recordings of the anonymized interviews (n=23) were analyzed by the first author (RA). Thematic analysis was applied, inspired by Brown and Clark [92], using NVivo (QSR International) qualitative analysis software. End user needs were identified through in-depth analysis and inductive coding of the data until saturation was reached [92]. A second researcher (MS) analyzed 17% (4/23) of the transcribed interviews for analysis validation. Overall, 2 researchers (RA and MS) participated in all of the coding of the identified needs into value categories (performed independently of each other), and a third researcher (JW) participated in validating 1/3 of the coding. The findings were continuously discussed within the research team during this process (RA, LN, MS, JW, LP, and JH). All steps contributed to the development of a comprehensive understanding of the data and to identify a representative and specific set of end user values and needs, as expressed by the key stakeholders [75,76,93]. Multimedia Appendix 3 presents examples from the analysis process related to the key values.

#### Focus Groups

The design elements and related needs identified during the 3 focus group sessions were analyzed, documented, and combined by the first author into one overview in a mind mapping tool (XMind, XMind Ltd) [94]. The findings were discussed with the service designer, digital designer, and the Scrum product owner participating in the focus groups, next to the research team. The identified design elements served as input for the analysis and identification of PSD principles and BCTs to meet end user values and needs (Figure 3).

#### Identifying PSD Principles, BCTs, and Design Requirements

To identify PSD principles and BCTs, the PSD model by Oinas-Kukkonen [26], behavior change taxonomy by Michie [43], previously conducted scoping review [23], and design elements from the focus group sessions were employed (Figure 3). Through small iterations in the analysis process, needs could be linked to one or more end user values. These could then be linked to possible PSD principles and BCT clusters, leading to the formulation of high-level requirements and suggested design features. The identification analysis was performed by the first author and one of the coauthors (RA and MS). A third coauthor (JW) validated 1/3 of the analysis with regard to inconsistencies and disagreements (randomly selected) and contributed to the requirement development. Data analysis and requirement development were continuously discussed by the research team. This identification process resulted in an overview of PSD principles and BCTs aimed at supporting end user values and needs as well as high-level requirements of an eHealth intervention aiming to support weight loss maintenance. The high-level requirements will allow for innovation during the next steps of the design and development process (Figure 1).

## Results

### Key Stakeholders and Demographics

#### Key Stakeholders

**Figure 4:**
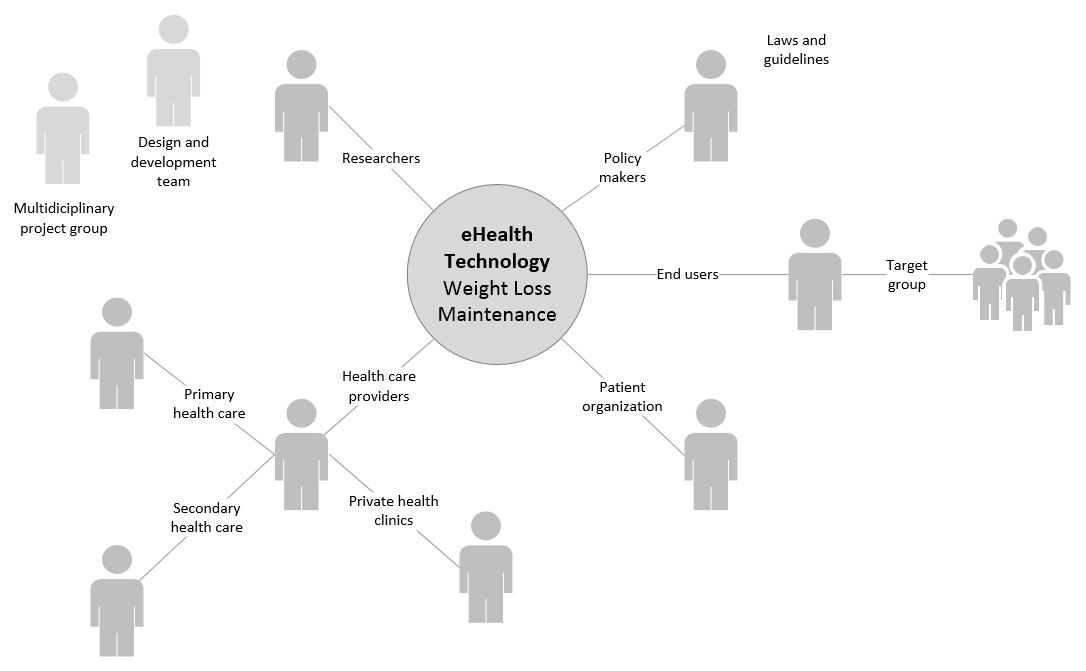
Key stakeholder map.

The key stakeholders identified were prospective end users (ie, people with obesity aiming to maintain weight after initial weight loss) and health care providers from primary and secondary health care providing health services to the target group. Additional key stakeholders identified were policy makers (ie, from the Norwegian Directorate of eHealth); representatives from a patient organization (ie, the Norwegian Association for People with Obesity); and researchers conducting research in behavioral medicine, obesity, and weight loss maintenance with clinical experience. See Figure 4 for a final key stakeholder map. In addition, the multidisciplinary design, software, and content development teams involved in the research project were consulted for expert opinions related to digital design and development (included as indirect stakeholders). Figure 2 presents an overview of key stakeholder involvement in each of the separate research processes in this study (N=50).

#### Prospective End Users Involved: General Characteristics

A total of 21 prospective end users, 67% (21/50) women, median age 53 years (range 24-70 years), participated in individual interviews and focus groups. As presented in Multimedia Appendix 4, approximately two-thirds of the participating end users reported having had obesity and weight maintenance challenges for most of their life. All end user participants reported having tried various diets (eg, low-calorie and/or very low–calorie diet) and/or conservative treatments (eg, lifestyle programs) to lose weight. They all also described having had several attempts to maintain their weight loss. All participants reported owning a smartphone, tablet, and/or computer and using them on a daily basis for various purposes (eg, social media, news, weather forecasts, financial services, and buying public transport tickets).

### Values and Needs of People Aiming to Maintain Weight After Weight Loss

**Figure 5:**
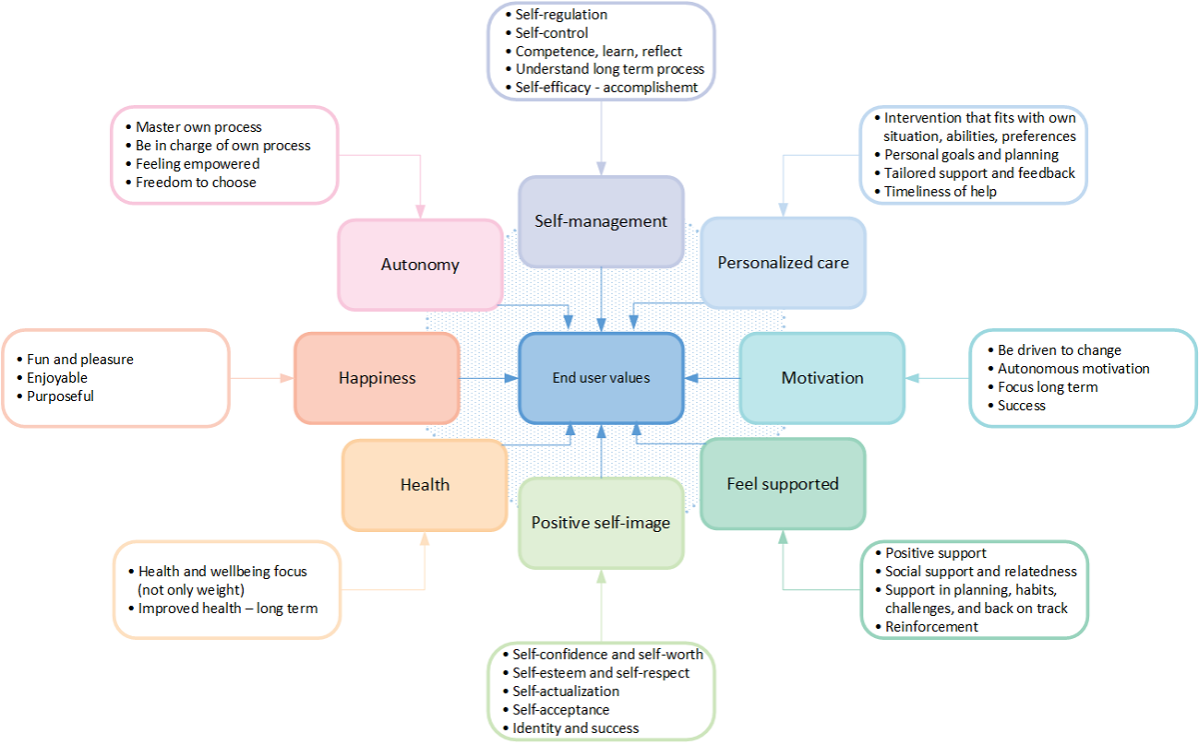
Overview of identified key end user values.

The results from the individual interviews with end users (n=11) and other key stakeholders (eg, health care personnel, presented in Figure 2; n=13) revealed that end user needs could be clustered into 8 themes (ie, key values) to maintain weight after weight loss. The 8 key values identified included *self-management*, *personalized care*, *motivation*, *feel supported*, *positive self-image*, *health*, *happiness*, and *autonomy*. See Figure 5 for details, including subcategories identified for each of the 8 interconnected values, with some of them overlapping one another.

Each specific key value (ie, high-level need or requirement) was generally supported by identified needs. Needs refer to demands and low-level requirements described by end users as aspects to be solved or met to address their weight maintenance problem. In this study, several needs were identified that underlie or support a specific key value (ie, high-level needs). In certain cases, a need could support multiple interrelated key values. For example, the need for *positive feedback on good and bad days to keep up with routines and healthy habits* could be linked to the value *motivation* and to the value *feel supported*. Similarly, the need for *personalized self-monitoring* could be linked to the value *self-management* and the value *personalized care*. Multimedia Appendix 3 provides an overview of examples indicating how *low-level* end user needs were connected to, and embodied, the key values. The following sections describe the findings related to each of the 8 identified key values.

#### Key Value: Self-Management

*Self-management* or *self-regulation* was one of the key values highlighted by participants as important to enable sustainable weight maintenance. Many people who struggle to prevent weight regain have difficulties regulating their eating behavior, engaging in physical activities, and establishing new habits:

Many lack competence and skills to do what they actually have learned in practice...

Health care personnel

In particular, self-regulation strategies and skills to implement into daily routines (eg, planning), dealing with challenges (eg, tempting situations and *back on track* when weight increases), and strategies for coping with emotional eating and impulse control were highlighted as essential needs within the concept of self-management:

Weight maintenance is all about planning. I would like to have different plans for different situations, a plan A for “normal days and weeks” and a plan B when “crisis occurs,” for example cake at work or holidays, to be able to withstand the constant feeling of hunger and tempting situations.

End user

For self-management, comprehension of one’s own process to create awareness and reflection around one’s own behavior was considered an essential need by end users and other key stakeholders:

To understand my own behavior and choices is really important. I can reflect and become aware of my own behavior.

End user

Help to identify automated behavioral patterns and unhealthy behaviors (eg, eating unhealthy food), self-monitoring of weight and healthy habit support is of essence to understand and regulate behavior...

Health care personnel

During the self-management process, the experiences of accomplishment, mastery, and success were deemed especially important to be able to “keep up” over time:

I try to focus on what I actually have reached so far. Sometimes I even try old trousers that are now too big...

End user

All stakeholders expressed the need for reliable and easily accessible information. As stated by one of the end users, “a lot of information is available for weight loss, but not for weight maintenance.”

#### Key Value: Personalized Care

All stakeholders emphasized that needs and requirements differ depending on the person and whether the focus is on weight loss or weight gain prevention. The differences described included aspects such as knowledge, support, and differences in self-management strategies to regulate eating behaviors, negative thoughts, and/or emotions:

People differ, and they experience different challenges over time.

Health care personnel

Tailored support to reach goals and establish healthy habits, including small personal steps (ie, subgoals), and to get personal feedback and “information that fits me” (end user) were other needs expressed within the personalized care value:

I would like to choose my own habits, goals and sub-goals to work on, such as reaching a weight target, eating more fruit or walk 6000 steps per day, and to keep track of my own progress.

End user

#### Key Value: Motivation

Finding motivation to keep up with the new habits was highlighted by all stakeholders as an important factor to *keep focus* and maintain weight. End users stated that being able to identify individual *motivators* or *reasons* (ie, intrinsic motivation) for maintaining weight and being able to select goals related to individual purpose were essential for staying motivated over time. Other key stakeholders described being able to focus on important purposes, defined by the end users themselves, could contribute to “strengthening of self-regulation capacity and the autonomous motivation required” for the necessary processes of change:

The real goal is to find out who you want to be and how you want to live your life, and set your own targets, what’s important to you?

Health care personnel

Examples of personal *motivators* described by end users included having good health, being more social, being able to play with grandchildren, being able to work, fit into *normal clothes*, and feeling confident. Receiving tailored (social) support and confirmation were also described by end users as crucial to stay motivated during difficult times or when struggling to self-regulate. Other key stakeholders described aspects such as practicing new habits and realistic goal setting, instant reinforcements (ie, rewards, tailored feedback) related to goals and outcomes, reminders of past success, and information about positive versus negative aspects of weight maintenance or regain as being of essence for continued motivation.

#### Key Value: Feel Supported

Feeling supported, with positive feedback to keep focus and stay motivated to maintain weight, was seen as crucial by all stakeholders. Several end users mentioned that they lacked support in their social surroundings and/or avoided social settings as much as possible. They described that being able to receive confirmation and (social) support, the feeling of not being alone, and having support on *good and bad days* or in *crisis situations* were important needs to be met during weight maintenance:

It feels safe when I am part of something and get follow up. It would be nice to have a technology that supports, especially if the weight goes in the wrong direction, in tempting or crisis situations.

End user

Other important aspects mentioned by end users included positive reinforcement (eg, praise and self-selected rewards), *just-in-time* support (eg, tailored feedback and 24/7 availability), and the ability to learn from others by sharing experiences with people in similar situations (eg, social learning, social comparison, cooperation, and social facilitation). These statements were supported by other key stakeholders:

It is really important that the technology motivates and supports me to keep up…

End user

They need a motivator, somebody cheering on the sideline, to be able to continue with the healthy habits to maintain weight.

Health care personnel

#### Key Value: Positive Self-Image

To feel confident and believe in oneself was mentioned by most stakeholders as being of essence for weight loss maintenance. The participating end users also stated that the focus should be on the whole person, not solely on their weight:

I want to be seen as a person.

End user

Several end users and other key stakeholders described that many have low self-confidence after several attempts at weight reduction:

Repetition is the clue and regular confirmation, to not fall back into old routines. If they fall back, they lose their confidence even more.

Health care personnel

Positive focus on one’s strengths and capabilities, the experiences of success, respect for oneself, identity, and self-worth were other areas identified by all end users as important to address to create a more positive self-image and support self-actualization:

I feel confident when I know that I do the right things to stay on track.

End user

#### Key Value: Health

Several end users described that the cost of maintaining weight after weight loss tends to outweigh the benefits. End users, therefore, stated that they not only want to focus solely on weight but also on positive health effects of a healthier lifestyle and of being less heavy. They indicated that *having a good health* was one of their main initial motivators to lose weight. Other key stakeholders highlighted aspects such as focusing on short- and long-term health effects of target behavior as important factors to support a feeling of enhanced control and improved health and well-being:

We try to focus on their health and well-being—on the positive effects—some still feel the costs more than the benefits.

Health care personnel

#### Key Value: Happiness

The end users expressed a preference to bring attention to enjoyable, fun, and positive benefits of healthy behaviors:

Healthy diet and good health should be something fun and nice, not something that gives me a bad conscience and demands a lot of energy.

End user

To make physical activities more fun, several end users reported using activity trackers or smartphones to monitor activity levels.

Many are highly motivated in the beginning of the weight maintenance phase, until they notice they are still hungry and start to struggle with the same things as in the past. To stay motivated, finding physical activities that bring joy or added purpose and are worth continuing can help.

Health care personnel

In terms of technology, special attention was also directed toward fun and enjoyable elements to stimulate happiness and positive feelings, so that a healthy lifestyle could be followed to maintain weight:

A technology that gives a fun and meaningful experience...I like to get inspired in a positive and fresh way, maybe with colors, illustrations, symbols and movies.

End user

The stakeholders also described being able to focus on short- and long-term health rewards of daily habits, through monitoring and rewarding healthy behaviors or targets, as ways to increase pleasure and happiness. One health care personnel said, “A technology that gives joy and a sense of meaning or added value will be used.”

Suggestions and information to make healthy eating more attractive and tastier were also described as ways to increase pleasure and happiness. Other key stakeholders also described factors such as making healthy eating more attractive and enjoyable, being able to get information on the health benefits of being active, and to see body composition improve as important factors for gaining insight.

#### Key Value: Autonomy

Many end users described the freedom to choose and be in charge as essential aspects to independently master their own behavior change process. They stated that mastery could be reached by having knowledge, abilities, and skills to achieve a healthy lifestyle and continue to do so. The end users also expressed that having customized plans (eg, for weekdays, weekends, holidays) would help induce a feeling of free choice, mastery, and control. Other key stakeholders also supported this:

I would like several modules, not dependent on each other...menus to choose from. A personal choice of what to register, which data to share, and when.

End user

I think the user should be able to choose their own functions and focus...some need more focus on diet, while other need structuring and planning, like a calendar or reminders.

Health care personnel

All stakeholders described the following factors as important to be able to choose and continue with healthy habits: facilitating a better understanding of the end users’ own process and behavior, enhancing the understanding of how the body and mind work, and learning about health and strategies for self-regulation:

It is important to realize that you have a choice. It is not the surroundings that decide what you eat or do.

Health care personnel

### Identified PSD Principles, BCTs, and Design Requirements of an eHealth Weight Loss Maintenance Intervention

#### Focus Group Results: Design Elements

Focus group findings provided an overview of potential *design elements* that could meet end user needs when aiming to maintain weight after weight loss. See Table 1 for details. The findings indicate that some design elements were considered more important to incorporate in eHealth weight loss maintenance interventions than others. However, most design elements were characterized as *must have* during focus groups 2 and 3.

**Table 1:** Focus group results with identified design elements.

Design elements (cards)	Focus group 1 (end users)	Focus group 2 (end users)	Focus group 3 (health care personnel)
Goal setting	++^a^	++	++
Planning	++	++	++
Motivating messages	++	++	++
Personalization	++	++	++
Self-monitoring	++	++	++
Visualization	++	++	++
Tailoring	++	++	++
Feedback	++	++	++
Knowledge^b^	++	++	++
Decision support^b^	++	++	++
Suggestions	++	+^c^	++
Rewards	+	++	++
Reminders	+	++	++
Wearables or sensor technology	+	++	++
Practice habits	+	++	++
Rehearse on situations or challenges	+	++	++
Social support: health care personnel	+	++	++
Social support: other users	+	+	++
Social support: virtual coach	+	−^d^	+
Gamification elements	−	+	+
Metaphors	−	−	−
Avatar	−	−	−

^a^Elements characterized as *must have.*

^b^Design elements suggested by focus group participants.

^c^Elements characterized as *nice to have.*

^d^Elements characterized as *not needed*.

Needs identified during open brainstorming in the focus groups were linked to the design elements. See Table 2 for details. In the analysis process examining the results, the 8 key values were linked to the needs identified.

**Table 2:** Focus group results with design elements to support weight maintenance needs.

Key values								Needs	Design elements cards (PSD^a^ and/or BCT^b^)
V1^c^	V2^d^	V3^e^	V4^f^	V5^g^	V6^h^	V7^i^	V8^j^		
✓^k^	—^l^	—	—	—	✓	✓	✓	Set own goals (personalized) Subgoals (eg, eat more fruit)	Goal setting: BCT—goals and planning
—	—	—	—	—	—	✓	✓	Build strategies and plans for different situations (eg, weekdays, weekends, holidays, and birthdays) Crisis plan and support plan (eg, on “bad days” or periods) Back-on-track strategy (eg, when drawback occurs, weight increases)	Planning: BCT—goals and planning
✓	✓	—	—	—	✓	—	—	Positive or motivational messages: Personalized feedback when reaching goals or achievements (eg, self-selected rewards, motivational words, and reminders of personal drivers for losing weight) On a “difficult” day or period When using the app (eg, “Welcome back! What’s the status?”) Show positive health effects No negative feedback	Motivating messages: PSD—praise BCT—feedback and monitoring
✓	—	—	—	—	✓	✓	—	Personal goals, monitoring, plans, and reminders Personal messages (praise) and self-selected rewards Several modules, interdependent (personal choice)	Personalization: PSD—personalization
—	—	—	—	—	—	—	✓	Long-term monitoring of behavior, goals, and plans (eg, through visualizations) My diary (easy and quick self-selected registrations)	Self-monitoring: PSD—self-monitoring BCT—feedback and monitoring
—	—	—	—	—	✓	—	✓	Understand own behavior: holistic insights health and well-being (eg, weight, activity, emotion, sleep, and stress)	Visualization: BCT—feedback and monitoring
✓	—	—	—	—	—	—	—	Smart, tailored feedback related to individual lifestyle, goals, and behavior (eg, automatic activity trackers) Automatic adaptation: favorite modules and interests easily available	Tailoring: PSD—tailoring
—	✓	✓	—	—	✓	—	✓	Health promoting (eg, positive spin-offs of maintaining weight and healthy behavior) Positive “boost” messages related to behaviors or habits Positive feedback on “bad days” (eg, personal values, positive goal focus, and earlier achievements) Tailored support (eg, related to crisis plan, tips and feedback, and timely) Smart, tailored feedback-based automatic monitoring and registrations	Feedback: PSD—praise BCT—feedback and monitoring
—	—	—	—	—	✓	✓	✓	Competence and skills to regulate behavior, emotions, and thoughts Information: How the body works after weight loss and recent research and knowledge to maintain weight Effects and benefits of healthy behaviors and maintaining weight (eg, fun facts) Preferably presented through a choice of text or sound, supported by visuals and movies	Knowledge: BCT—shaping knowledge and natural consequences
—	✓	✓	—	—	—	—	✓	Reflect on behaviors and decisions Support in making healthy choices	Decision support: PSD—reduction and suggestions BCT—antecedents, goals and planning, (eg, action planning), and associations
—	—	—	✓	—	✓	—	✓	The best evidence-based strategies and tips to maintain weight Focus on health and well-being (eg, how to reach goals and keep up with healthy habits) Practical tips (eg, self-regulation, when technology is not enough)	Suggestions: PSD—suggestions BCT—regulation
—	—	—	—	✓	✓	—	—	Rewards (eg, points, trophies) related to goals and targets (eg, weight and activity goals) Self-selected rewards for motivation	Rewards: PSD—rewards
	—	—	—	—	—	—	✓	Reminders: My goals, values, and plan Healthy habits to maintain weight and how the body works When it goes well and when “off track” (eg, reminders of past successes, trouser that is too big, before and after pictures)	Reminders: PSD—reminders and simulation BCT—associations (ie, prompts and cues), self-belief and identity
—	—	—	—	✓	✓	—	✓	Automatic registrations and automatic behavior trackers (eg, weight, activity trackers, wearables, sensors, and smart devices) Ease of use, easy monitoring, and long-term storage of data Personal contact or helper (eg, family or friend for motivation and support)	Wearables or sensor technology: PSD—self-monitoring BCT—feedback and monitoring (ie, biofeedback)
—	—	✓	—	—	—	✓	✓	Practice (new) healthy habits Keep up with daily routines and healthy habits in the long term	Practice habits: PSD—reduction and rehearsal BCT—repetition and substitution (ie, habit formation)
—	—	✓	—	—	—	✓	✓	Train and prepare for risk situations or tempting situations (eg, “what-if plans,” impulse control, and self-regulation)	Rehearse on situations or challenges: PSD—rehearsal BCT—repetition and substitution and goals and planning
—	✓	—	—	—	✓	—	✓	Contact with coach or health care personnel or general practitioner (eg, when technology is not enough) Support or personal helper (eg, family, friend, or other users or peers through social forum or chat or inspirational user stories) to share experience, learn about health-related behaviors from others, and cooperate	Social support: PSD—social learning, social cooperation, and social facilitation BCT—social support and comparison of behavior
—	—	—	—	✓	✓		—	Points and trophies when reaching goals and targets to keep focus and motivation Animated coach for motivation and joy	Gamification elements: PSD—rewards BCT—reward and threat

^a^PSD: persuasive system design.

^b^BCT: behavior change technique.

^c^V1: personalized care.

^d^V2: feel supported.

^e^V3: positive self-image.

^f^V4: health.

^g^V5: happiness.

^h^V6: motivation.

^i^V7: autonomy.

^j^V8: self-management.

^k^The value was highlighted by the focus groups based on the needs and related design elements identified.

^l^The value was not highlighted by the focus groups based on the needs and related design elements identified.

The findings indicate that several design elements, supported by PSD principles and BCTs, can be applied to facilitate and motivate short- and long-term behavior changes. Positive *feedback, associations* (eg, prompts and cues), and rewards (eg, earning points and receive trophies) were identified as motivational elements to inculcate new behavior and for short-term goal setting during the focus group sessions. For long-term behavior change, rewarding strategies linked to, for example, *self-selected rewards*, *identity* (eg, reminders of past successes), *natural consequences* (eg, information about health consequences of performing the behavior), and *social support* (eg, social cooperation and social learning) were elements identified as potential motivators for sustainable change.

The findings presented in Tables 2 and 3, supported by findings from the previously mentioned scoping review [23], served as input in the analysis to identify PSD principles, BCTs, and requirements of an eHealth intervention to support end user values and needs.

**Table 3:** Identified behavior change technique clusters supporting end user values.

Behavior change technique clusters based on behavior change taxonomy by Michie	Key values							
	V1^a^	V2^b^	V3^c^	V4^d^	V5^e^	V6^f^	V7^g^	V8^h^
Scheduled consequences	—^i^	—	—	—	—	—	—	—
Reward and threat^j^	—	—	—	—	✓^k^	✓	—	—
Repetition and substitution^l^	—	✓	—	—	—	—	✓	✓
Antecedents	—	—	—	—	—	✓	✓	✓
Associations^j,l^	—	—	—	—	—		✓	✓
Covert learning	—	—	—	—	—	✓	—	—
Natural consequences^m^	—	—	—	✓	—	✓	✓	✓
Feedback and monitoring^j,l,m,n^	✓	✓	✓	✓	✓	✓	✓	✓
Goals and planning^j,l,m,n^	—	✓	—	—	—	✓	✓	✓
Social support^j,l,m,n^	—	✓	✓	—	—	✓	—	✓
Comparison of behavior	—	—	—	—	—	—	—	✓
Self-belief^m^	—	—	✓		✓	✓	✓	✓
Comparison of outcomes	—	—	—	—	—	✓	—	—
Identity^m^	—	—	✓	—	✓	✓	✓	—
Shaping knowledge^l,m,n^	✓	—	—	✓	—	—	✓	✓
Regulations^m^	—	—	✓	—	✓	✓	—	✓

^a^V1: personalized care.

^b^V2: feel supported.

^c^V3: positive self-image.

^d^V4: health.

^e^V5: happiness.

^f^V6: motivation.

^g^V7: autonomy.

^h^V8: self-management.

^i^Not identified BCTs supporting key values.

^j^Behavior change techniques (BCTs) mentioned applied to stimulate motivation and/or adherence in weight loss maintenance interventions (for long-term change), identified in the previously performed scoping review [23].

^k^Identified BCTs supporting the key values.

^l^Most frequently applied behavior change techniques in weight loss maintenance interventions, identified in the previously performed scoping review [23].

^m^The most frequently identified behavior change techniques in relation to key values in this study.

^n^BCTs included in eHealth interventions that found significant effects for weight loss maintenance, identified in the previously performed scoping review [23].

### Identified PSD Principles, BCTs, and Design Requirements of an eHealth Intervention to Support End User Values and Needs

The end user values and needs identified were mapped using BCT taxonomy by Michie [43] to identify BCT clusters of potential relevance for eHealth interventions supporting long-term weight loss maintenance. See Table 3 for the results of this mapping and analysis.

Of the 16 BCT clusters, 15 could be linked to the identified 8 key values for eHealth weight loss maintenance interventions. Most BCTs were linked to the key values *self-management* (11/16), *motivation* (11/16), and *autonomy* (9/16). BCT clusters supporting 4 key values or more included *natural consequences* (eg, information about health and emotional consequences), *feedback and monitoring* (eg, related to weight and behavior), *self-belief* (eg, focus on past success and positive self-talk to raise self-confidence), *goals and planning* (eg, goal setting, action planning, and problem solving, including relapse prevention and coping planning of risks for weight regain), *identity* (eg, focus on personal strengths or purpose for behavior change associated with the new behavior), *shaping knowledge* (eg, advice and strategies related to diet, physical activity, and behavior change), *regulations* (eg, skills to regulate or reduce negative emotions), and *social support* to maintain healthy behaviors.

As indicated in Table 3, *goals and planning*, *feedback and monitoring*, and *social support* were identified as important for weight loss maintenance support by the analysis of BCTs to meet end user values as well as findings from the previous scoping review [23]. The BCT clusters *self-belief*, *natural consequences*, and *identity* were also highlighted as being of essence to meet end user values. However, in the previous scoping review, these BCT clusters were not recognized as frequently applied techniques [23]. The identified BCT clusters in Table 3 (including the underlying techniques) were considered during the high-level requirements specification to illustrate possible combinations of BCTs and PSD principles. Multimedia Appendix 3 presents examples of the BCTs in relation to values, needs, and high-level requirements.

To map the PSD principles relating to end user values and needs, the PSD model [26] was applied. See Table 4 for details. The findings indicate that most of the identified PSD principles were from the *primary task* (6/7) and *dialog support* (6/7) categories of the PSD model [26], followed by the *social support* (4/7) and *credibility support* (4/7) categories.

**Table 4:** Identified persuasive system design principles supporting end user values.

Persuasive system design principles		Key values							
		V1^a^	V2^b^	V3^c^	V4^d^	V5^e^	V6^f^	V7^g^	V8^h^
**Primary task support**									
	Reduction	✓^i^	—^j^	—	—	—	—	✓	✓
	Tunneling	—	—	—	—	—	—	—	—
	Tailoring^k,l,m^	✓	✓	✓	✓	✓	✓	✓	✓
	Personalization^k,l,m^	✓	✓	✓	✓	✓	✓	✓	✓
	Self-monitoring^k,l,m,n^	✓	—	—	✓	✓	✓	✓	✓
	Simulation^l^	✓	—	—	—	—	✓	—	✓
	Rehearsal	—	✓	—	—	—	—	✓	✓
**Dialog support**									
	Praise^k,m^	✓	✓	✓	✓	✓	✓	—	✓
	Rewards^m^	✓	—	—	—	✓	✓	—	—
	Reminders^l^	✓	—	—	—	—	✓	—	✓
	Suggestions^k^	—	✓	—	✓	—	✓	✓	✓
	Similarity	—	—	—	—	—	—	—	—
	Liking	—	—	—	—	✓	—	—	—
	Social role	—	✓	—	—	—	✓	—	—
**System credibility support**									
	Trustworthiness	—	—	—	✓	—	—	—	✓
	Expertise	—	—	—	✓	—	—	—	—
	Surface credibility	—	—	—	—	—	—	—	—
	Real-world feel	—	✓	—	—	—	—	—	—
	Authority	—	—	—	—	—	—	—	—
	Third-party endorsements	—	—	—	—	—	—	—	—
	Verifiability	—	—	—	✓	—	—	—	—
**Social support**									
	Social learning	—	—	—	—	—	✓	—	✓
	Social comparison	—	—	—	—	—	✓	—	✓
	Normative influence	—	—	—	—	—	—	—	—
	Social facilitation	—	—	—	—	—	—	—	✓
	Cooperation	—	✓	—	—	—	—	—	—
	Competition	—	—	—	—	—	—	—	—
	Recognition	—	—	—	—	—	—	—	—
**Others^o^**									
	Feedback^k,l,m,n^	✓	✓	✓	✓	✓	✓	—	✓
	Goal setting^k,l,m,n^	✓	—	—	—	—	✓	✓	✓
	Social support^k,m,n^	—	✓	✓	—	—	✓	—	✓

^a^V1: personalized care.

^b^V2: feel supported.

^c^V3: positive self-image.

^d^V4: health.

^e^V5: happiness.

^f^V6: motivation.

^g^V7: autonomy.

^h^V8: self-management.

^i^Identified PSD principles supporting the key values.

^j^Not identified PSD principles supporting the key values.

^k^The most frequently identified persuasive system design principles in relation to key values in this study.

^l^Most frequently applied persuasive system design principles in weight loss maintenance, identified in the previous scoping review [23].

^m^Persuasive system design principles mentioned applied to stimulate motivation and/or adherence in weight loss maintenance, identified in the previous scoping review [23].

^n^Persuasive system design principles included in eHealth interventions that found significant effects for weight loss maintenance, identified in the previous scoping review [23].

^o^Persuasive principles added from the scoping review[23].

PSD principles supporting 4 or more key values included *personalization* (eg, of goals, self-selected rewards, and content), *tailoring* (eg, of feedback and information), *praise* (eg, positive, motivating feedback to keep up with healthy behavior), *self-monitoring* (eg, of behavior that supports reaching goals), *feedback* (eg, tailored in relation to already accomplished goals, outcomes, and behavior), *suggestions* (eg, about healthy eating habits), *goal setting* (eg, of outcome and target behavior), and *social support* (eg, through social learning, social comparison, cooperation, or social facilitation by humans or a virtual coach and/or peers). Multimedia Appendix 3 presents examples of the PSD principles identified and how they can be combined with BCTs when specifying high-level requirements for persuasive eHealth technologies supporting weight loss maintenance.

The key values presented under the *Values and Needs of People Aiming to Maintain Weight After Weight Loss* section and in Tables 3 and 4 represent the wants of the end users and their reasons for those wants. The identified BCT clusters and PSD principles indicate techniques that can be applied to change behavior to maintain weight. Many of the BCT clusters, including underlying techniques, were identified as important to address long-term behavior change (eg, *habit formation*). The requirement specification included in Multimedia Appendix 3 shows examples of how BCT clusters, such as *repetition and substitution,* can be combined with PSD principles such as *suggestions* and *reduction*, to support sustained behavior change through eHealth technologies.

## Discussion

### Principal Findings

This study aimed to provide insight into the design of eHealth interventions supporting behavior change for long-term weight loss maintenance. The goal was to identify values and needs of people with obesity aiming at maintaining weight after weight loss, and to identify PSD principles, BCTs, and design requirements that meet these values and needs in eHealth interventions. The results revealed specific interconnected key end user values (ie, main drivers of behavior or high-level requirements) and needs (ie, demands or low-level requirements), and also provided insight into BCT clusters and PSD principles and how they can be combined to support end user needs in eHealth interventions.

#### End User Values and Needs to Maintain Weight After Weight Loss

The findings from the semistructured interviews and focus groups with key stakeholders were consistent and complemented each other with respect to the 8 interconnected key values that were identified in this study: *self-management, personalized care, motivation, feel supported, positive self-image, health, happiness, and autonomy.* The key values (ie, main drivers of behavior or high-level requirements) were each supported by identified needs (ie, demands or low-level requirements), sometimes supporting multiple key values. The identified values were interconnected, rather than strictly separated, and dynamic, as the drivers and individual needs can change during long-term maintenance of lost weight.

The 8 identified key values may drive the need for behavior change that is required to successfully maintain weight over time, and motivation and chance for success may increase as these values are met. This means that in eHealth interventions aiming to facilitate sustainable weight loss maintenance, supporting the identified key values in this study could be of essence.

Several of the overlapping end user values identified in this study are in line with research and theories arguing that *most behavior is multi-motivated* and that a value-driven and positive approach to health and well-being is required to achieve sustainable behavior change [95-98]. In such a holistic approach, behavior tends not to be determined by one value or specific goal but by several dynamic needs simultaneously [96,97,99,100]. Theories about motivation, self-regulation, and social learning [101-104] are likely to be of use in the design of eHealth interventions emphasizing values and needs, as identified in this study. For example, the self-determination theory [103,105] offers a macro theory of human motivation and personality, suggesting that motivation to change is dependent on whether people’s needs for competence, autonomy, and social relations or relatedness are met. A such, self-determination theory could potentially act as a framework to support several of the identified values and needs, including the individual needs for knowledge and skills (ie, competence) to self-manage, and individual differences with regard to personality, psychological, and/or motivational profile [103,105-108].

#### PSD Principles, BCT Clusters, and Design Requirements to Meet End User Values and Needs

Several PSD principles and BCT clusters were identified as potentially promising to meet the identified key end user values. Findings from this study are consistent with the results from the scoping review previously conducted by the research team [23], indicating that *goals and planning*, *feedback and monitoring*, and *social support* should be pursued long term to maintain weight [22,23,107,109-114]. However, this study revealed some BCT clusters not previously identified (eg, *identity* and *self-belief*) [23] that could be important to maintain long-term weight loss.

The challenge related to weight loss maintenance for people with obesity is evident, as many people struggle to maintain weight and may easily fall back into old habits following weight loss [6]. This can be explained by the nature of habits, as habits are often learned, automatic, and sometimes even unconscious processes formed through repetition [25]. In this study, BCTs within the *repetition and substitution* clusters (eg, *habit formation* and *habit reversal*) were identified. *Habit formation* can be integrated into eHealth interventions to support the formation of new and lasting habits and might be a missing link in the effort to address the challenges related to weight loss maintenance [7,10,14,15,22,115-121]. To adapt to the dynamic changes in individual needs over time and to support contextual changes, *personalization* and *tailoring* were identified PSD principles of essence in this study.

Findings from this study point to a combination of several PSD principles and BCTs as a necessity to meet end user values and needs in eHealth weight loss maintenance interventions. Persuasive technologies and application of PSD principles in digital behavioral interventions might be key to support long-term weight loss maintenance, especially when combined with BCTs and behavior change theories [23,47]. However, evidence and guidance on how to combine these strategies are nonexistent [23]. PSD principles can be applied to create engaging, user-friendly, persuasive technologies that can support healthy choices and decisions and may be particularly important in *the adoption of new habits* (eg, *suggestions*, *reduction, self-monitoring, rewards,* and *feedback*). On the other hand, BCTs seem to be important for sustainable behavior change, and the identified techniques (eg, *goals and planning*, *monitoring and feedback*, *repetition and substitution*, *associations*, *antecedents, regulation of emotions*, *self-belief*, and *identity*) can be learned and put into daily life, even without technology.

In this study, for some of the key values, only a few supporting BCT clusters and/or PSD principles could be identified. This could indicate that some BCTs or PSD principles have yet to be discovered, possibly because some needs may be future or latent needs. It could also indicate that BCT clusters (eg, *covert learning*) [43] that are not fully desired through identified end user values or needs, but could none the less be important for the required behavior change, should receive particular attention in technology design and may need to be combined with PSD principles (eg, *rehearsal*) [26] to support the user and create the aspired impact on behavior change.

#### Implications for Future Design and Practice

There are numerous potential advantages of digital health technology. Digital technologies can, for example, overcome time and place barriers, with improved accessibility (24/7 availability) and timely support; enable people to track and aggregate real-time data from sensors and smart devices; and may also support personalized and tailored interventions, depending on user behaviors and needs [122]. To date, however, research examining the design for sustainable behavior change is still sparse [23,123].

If centered around end user needs and priorities, digital self-management support systems have the potential to meet several of the key values identified in this study [104,124-128], which might lead to the long-term behavior change needed to maintain weight. The success of such systems also depends on the incorporation of facilitating factors linked to weight maintenance, including self-regulation strategies (eg, cognitive and emotion regulation, goal setting, effective coping, and problem-solving skills), and increased confidence in own ability to self-regulate [13,104,126-128].

Reversal of established behaviors and habits can be challenging, particularly over time [129,130]. However, applying a combination of the identified BCTs and PSD principles (eg, *repetition and substitution, identity, feedback and monitoring, goals and planning,* and *personalization*) in future eHealth interventions, linking healthy habits to self-determined goals and identity (eg, individual purpose, health or life goal), might contribute to sustainable behavior change and long-term weight control [52,105,108,125,129,131-136]. According to end users and other key stakeholders, many experience that it costs more than it benefits to change behaviors for continued weight control after weight loss. As identified in this study, digital technologies that are designed to support personal and autonomous motivation, self-efficacy, self-regulation skills, positive body image, self-selected rewards, and health and well-being (eg, not only weight) could also contribute to long-term weight control and improve the *cost-benefit* ratio that many people experience while trying to balance behaviors to prevent weight regain [23,103,104,111,125,131,134,137,138].

Although eHealth technologies can contribute to improve health care, support sustainable behaviors promoting health and well-being [139], and increase impact and uptake through personalized and tailored design [36,45,122,140-142], eHealth interventions can only be effective if actually used. This means that persuasive and engaging technologies, as examined in this study, have the potential to support people in engagement and adherence to healthy lifestyles in pursuit of their goal of weight maintenance [30,34].

Behavior change interventions are usually complex, and some studies show how interventions incorporating multiple BCTs, rather than just a few BCTs, tend to have larger effects promoting health behavior change [43,54,143-145]. Digital behavioral obesity interventions that combine PSD principles with behavior change theories have also been shown to produce statistically significant weight loss results more frequently [47]. Specifying one *ideal* combination of PSD principles and BCTs to support sustainable behavior change and weight control in eHealth interventions might not be possible. Varying individual values and needs, which requires a personalized and tailored approach, make specifying an *ideal* combination at the very least challenging [15,142,146,147]. Investigating which PSD principles, BCTs, and content that is most effective for sustained engagement, for whom and in which context, should be a goal for future research developing or examining digital weight loss maintenance interventions [107].

With several biological, behavioral, psychological, social, and environmental factors interacting, the challenges related to obesity and weight loss maintenance are complex and numerous [16,21]. In this study as well as in the scoping review previously published by the research team [23], personalization and tailoring, including tailored support and personal feedback, were highlighted as factors of great importance for the success of digital interventions targeting behavior change and weight loss maintenance. Individual differences, including personality and psychological profiles that may impact behavior and behavior change, should also be examined and taken into consideration when designing and tailoring such digital interventions in the future [106]. Tailoring based on individual characteristics (eg, personality, motivational orientation) could be of essence when aiming to create effective technological features supporting behavior change [142]. Cognition, affect, and behavior related to individual context have also been identified as core components of engagement and should be considered in the design and development, including the form of delivery and content, of digital interventions aiming to enhance and sustain engagement and support health behavior change [107,147]. In the development process, this issue could be addressed by identifying a variety of user types (eg, personas) [148], and the combination of applied PSD principles and BCTs may potentially be adjusted or *tailored* per user type [142,149]. Ideally, BCTs and PSD principles applied in eHealth behavior change interventions should allow for flexible interaction and adaptability based on the dynamic and changing individual end user needs over time.

Rapid technology developments have changed the way modern organizations approach innovation to create value for end users and stakeholders [68,69,91,150-152]. This study integrates design thinking with BCTs and PSD principles and can guide co-design processes to identify design elements to meet end user values and needs for behavior change in future interventions [64,65,153]. The proposed method used in this study also unlocks and explores current, latent, and future user needs and possible solutions through its iterative and participatory process [21,39,40,68-72,77,154]. The key values, BCTs, and PSD principles identified in this study can provide input to future design and development processes of eHealth weight maintenance interventions. Co-design and prioritization of BCTs and PSD principles with key stakeholders, including designers and developers, may further develop and specify eHealth interventions, allowing for functional requirements and physical design to be adapted and optimized during prototype development and user testing [40,73,91,155]. In addition, multidisciplinary design teams and quick experiments during the entire design and development process may increase the chance for the technology design to successfully meet user needs. However, as shown in this study, some values and needs could be conflicting. The findings from this study also suggest that some needs, PSD principles, or BCTs for sustainable behavior change are yet to be discovered [23,36].

### Recommendation for Future Design, Research, and Implementation

Considering the significant individual and public health challenges of obesity and weight maintenance after weight loss, novel, human-centered, and evidence-based solutions aiming to close the gap between weight loss and maintenance of the new weight for long term are needed. First, future design and development of research-based eHealth interventions targeting weight maintenance should aim to investigate how to meet the key values identified in this study and capture expectations and uncovered, or potentially latent, end user needs to support weight maintenance over time [91]. Second, personalization appears to be an important ingredient in successful behavior change. Future research should, therefore, examine the role of personalized and emerging eHealth technologies, supporting the integration of identity-oriented approaches with habit formation and self-regulation strategies to achieve long-term behavior change needed to maintain weight after loss. Third, design elements that facilitate autonomous motivation, create a positive experience and engage users over time, contribute to making healthy behaviors enjoyable, and improve self-efficacy and positive self-image through technology should also be examined [141,147,156].

People, technology, and context are intertwined [39,42,157]. Both services and technologies are shaped through their use, which is why design that allows for interaction and continuous adaptation to user requirements, through personalization and tailoring, is particularly important for adoption and long-term use. To be effective, future solutions aiming to support weight loss maintenance should therefore aim to fit both end user values and needs as well as intervention features and context [42,158]. For researchers, designers, and developers concerned with supporting long-term weight loss maintenance, the findings indicate potential PSD principles and BCTs that can be applied in digital design for sustained behavior change to address the identified key values. Future research should therefore investigate and evaluate how to combine the PSD principles and BCTs in the best way to develop effective, persuasive eHealth interventions supporting sustainable behavior change and weight loss maintenance. Evaluation of technology use also has the potential to reveal user profiles and promising PSD principles and BCTs, addressing the complexity of behavior change to meet individual needs to maintain weight over time [60,76,159]. The value proposition and technology features of future eHealth interventions can be further explored, prioritized, and specified through co-design, prototyping, and testing with key stakeholders, contributing to the next steps of technology design, development, and implementation.

### Strengths and Limitations

This study had some limitations. First, a few of the participating end users were still aiming to reduce weight, although their initial weight loss goal was achieved at the point of recruitment. However, this was the case for a few participants only, and as these participants were also focusing on weight gain prevention, the main results were not considered to be significantly affected. End user needs did differ during weight loss compared with weight maintenance, particularly with regard to dietary monitoring and control (ie, calorie count and registration). In reality, these aspects probably vary and interact. This study therefore likely reflects real-life aspects of weight loss or weight maintenance.

Second, more women than men participated in the study, which may have limited generalizability for the male population. Several other key stakeholders were male, potentially enhancing a representative perspective related to male needs and values when aiming to change behavior and maintain weight after weight loss, with the support of eHealth technology.

Third, not all participating end users had experience with successful long-term weight loss maintenance. Needs following a longer period of weight loss maintenance might be different from the needs following a short-term period. As most end users in this study were recruited within 2 to 3 months following weight loss, this could have influenced the findings in this study with regard to long-term needs. By including end users with positive as well as negative experiences from short- and long-term weight gain prevention, a broad view and understanding of the weight maintenance phenomena could be explored.

Fourth, the participants who volunteered for this study might have been more engaged, motivated, and interested in the use of eHealth technologies than the average weight maintenance key stakeholder. Given the nature of the qualitative methods applied in this study, the findings allow for detailed information about a complex issue. However, more research examining aspects of values and needs to maintain weight loss over time is required to enhance generalizability. Finally, identification of PSD principles and BCT clusters might have been prone to subjectivity by the researchers. However, to prevent subjective interpretation, 3 researchers participated in the validation of identified PSD principles and BCTs to reveal and address inconsistencies in this study.

This study also had several strengths. First, the research and development team were multidisciplinary, with clinical and research expertise within behavioral medicine, obesity, eHealth, and persuasive technology, and included user representatives as well as software developers and designers. Second, the methods applied required high levels of end user and other key stakeholder involvement. Such an approach can be time consuming but might be critical for success when designing and developing eHealth interventions [36]. Third, by applying a variety of qualitative methods and involving various stakeholders [36,75,93], a broad understanding of the issues at hand was achieved, capturing and verifying end user needs from various perspectives (eg, patients, health care personnel, and policy makers). Fourth, the mixed and converging research and design methodologies applied [39,68,72], involving multiple researchers in the data collection and analysis processes, aimed to increase validity and reduce possible researcher bias. Finally, the presentation of values and needs and a thorough description of the analysis process identifying and translating PSD principles and BCTs into high-level requirements aimed at contributing to transparency, understanding, and reproducibility of the findings.

### Conclusions

eHealth interventions have the potential to support the regulation of behaviors and maintenance of weight after weight loss. This study contributes to a better understanding of the values and needs of people aiming to maintain weight after weight loss. The translation of values and needs into design elements or features supported by PSD principles and BCTs could play an important role in the design of future eHealth interventions that support sustained behavior change and long-term weight maintenance. How PSD principles and BCTs can be combined in the best way to facilitate behavior change to achieve long-term weight loss maintenance remains to be determined.

The methods described in this study can guide the design of digital interventions and services supporting behavior change to meet end user values and needs in the future. To the best of our knowledge, this is the first study to present insights and suggestions for a new approach on how to identify and translate end user values and needs into PSD principles and BCTs when designing eHealth self-management interventions for sustained behavior change and weight loss maintenance.

## Appendix

Multimedia Appendix 1 Interview guide.

Multimedia Appendix 2 Design elements of feature cards.

Multimedia Appendix 3 Value specification, persuasive system design principles and behavior change techniques identification, and high-level requirements development for eHealth interventions supporting weight loss maintenance.

Multimedia Appendix 4 Infographics to visualize the demographics of included end users.

## Abbreviations

BCT: behavior change technique
CeHRes roadmap: Center for eHealth Research and Disease Management roadmap
OUH: Oslo University Hospital
PSD: persuasive system design
SH: Sørlandet Hospital
VHT: Vestfold Hospital Trust
