# Supplementary material for: Identifying Persuasive Design Principles and Behavior Change Techniques Supporting End User Values and Needs in eHealth Interventions for Long-Term Weight Loss Maintenance: Qualitative Study
Source: J Med Internet Res. 2020 Nov 30;22(11):e22598. doi: 10.2196/22598 (PMC7735908; doi:10.2196/22598)
Supplement: Multimedia Appendix 1 [file jmir_v22i11e22598_app1.pdf]

Multimedia Appendix 1

Interview Guide End users (ie, people with obesity aiming to maintain weight)

**Understand daily life, needs and coping strategies**	**Activities and routines**	Daily routines<br>A typical day (eating & physical activity behaviors, sleep, stress)
	**Good and bad days / periods**	Characteristics and causes
	**Challenges & problems everyday life**	Challenges or problems experienced in daily life
	**Coping strategies**	How do you deal with the challenges
		What works and does not work
	**Values in life**	Most important to you (highest priority)
		Most important reasons / Drivers to maintain lost weight
	**Motivation for change**	Motivation to change / lose weight
	**Experienced health**	Health and wellbeing before and after weight loss /new habits
	**Behavior change**	What was difficult with the behavior change / new habits
	**Understand the weight maintenance problem**	Could you describe:<br>- Everyday challenges<br>- The biggest challenges
	**Social support**	From family, friends, work
		From healthcare professionals / Healthcare system
	**Earlier treatment**	Earlier experiences and current needs
		Which needs have been met
		What do you miss or what could be improved
	**Needs behavior change and weight loss maintenance**	Unmet needs
		What is important for you with respect to health and weight
		What are the most important effects of the behavior change
**Weight loss maintenance**	**Preventing weight regain**	What have you done
		Most important strategies / actions
		How to manage / control impulses. What works and does not work.
		Useful knowledge / information
		New strategies that you learned, that worked
	**Motivation and behavior change**	How to stay motivated
		Experiences with continuing the new healthy habits / behaviors
		What has been most challenging (physical, mental, social)
		Difficult phases in the change process (back to old habits), what did you learn
		Eating behavior, physical activity, sleeping pattern, energy, challenges?
	**Needs to maintain weight**	What have worked / what helped you to succeed
		Self-management strategies
		Support from a person vs. technology to maintain changes / weight

**Technology**	**Technology experience and use**	Use of different technologies
		Use of technologies in relation to health and weight management (e.g., apps, trackers / wearables, blogs, internet)
		Why do you use this or not
		What makes you continue using it or not
		What do you like or don’t like with the solutions
		Communication with healthcare professionals or peers through technology
	**eHealth intervention weight loss maintenance**	Would an eHealth technology be useful (why/why not)?
		What would be useful to you / not useful?
		How can an eHealth technology help in your everyday life (what, when, where)?
		Content & presentation of content
		Design
		Needs for active use (look and feel)
		Privacy and security
		How much time would you spend on the technology?<br>Barriers / facilitators for use?
	**End of interview**	What is the most important to consider when starting the development process?
		Anything missing or that has not been mentioned?
		Other ideas or questions?

Interview Guide Other Stakeholders

**Understand patient / end-user challenges and needs**	**Own role and experience with the patient group.**	What and how
	**Main problems / challenges within the field.**	Why is this challenging? How can the problem be solved / challenges addressed?<br>Values and preferences.
	**Challenges & problems**	Challenges or problems in relation to health care provision, weight loss maintenance, behavior change (e.g., diet, physical activity, stress, sleep).<br>Stakeholders involved.
	**Needs behavior change and weight loss maintenance**	What do users need the most / unmet needs
		Key values and needs
	**Patient / end-user challenges**	Most important patient challenges (question for help)
		Most important reasons / Drivers to maintain lost weight
	**Weight loss maintenance offerings / support / services**	What can help end-users to maintain weight
	**How to improve health services to the patient group**	Current vs future offerings
	**Behavior change**	What do users experience as difficult with the behavior change / new habits / weight loss maintenance
	**Understand the weight maintenance problem**	Everyday challenges.<br>The biggest challenges users experience.
	**Preventing weight regain & long-term behavior change**	Most important strategies / actions
		How to manage / control impulses. What works and does not work.
		Useful knowledge / information
		Motivation

**Technology**	**Technology use and experience**	Use of different technologies
		Use of technologies in relation to health and weight management (e.g., apps, trackers / wearables, blogs, internet)
		Experience with use of technologies in health care provision
		Experience with use of technologies for weight loss maintenance
		What do you like or do not like with current solutions.<br>What can technology contribute with?<br>What do you wish?
		Communication with end-users / patients or other healthcare professionals through technology
	**eHealth intervention weight loss maintenance**	Would an eHealth technology be useful (why/why not)?
		In what way can an eHealth / digital technology help to sustain weight loss maintenance after initial weight loss? How?
		In what way can eHealth / digital technology support behavior change and continuation of healthy habits? How?
		Content & presentation of content
		Design
		Needs for active use (look and feel)
		Privacy and security
		How does the technology fit in the current service offerings?<br>Added value? Barriers / facilitators implementation?
	**End of interview**	What is the most important to consider when starting the development process?
		Anything missing or that has not been mentioned?
		Other ideas or questions?
