# Supplementary material for: Identifying Persuasive Design Principles and Behavior Change Techniques Supporting End User Values and Needs in eHealth Interventions for Long-Term Weight Loss Maintenance: Qualitative Study
Source: J Med Internet Res. 2020 Nov 30;22(11):e22598. doi: 10.2196/22598 (PMC7735908; doi:10.2196/22598)
Supplement: Multimedia Appendix 2 [file jmir_v22i11e22598_app2.pdf]

Multimedia Appendix 2 Design Elements Features Cards

Goal-setting
Set and follow own short and long-term goals

Game elements
Includes motivating & funny gamification elements

Planning
Plan e.g. one day, event, or specific period based on calendar function.

Motivating messages
Praise / positive messages on «bad days» or when almost reaching a target

Social support virtual coach
Provides support on difficult days, or when performing healthy habits / behavior!

Reminders
Reminders about targets or habits to work on

Wearables / sensors
Automatic tracking, measuring & registration

Practice habits
Train on new & just established habits, to learn new routines & make healthy choices

Suggestions
Tips on healthy habits and strategies, e.g., diet, activity, what to do when hungry

Train on situations / challenges
Support training on «tempting» / difficult situations

Avatar
Make own avatar based on oneself or preferences

Personalization
Personal preferences: look and feel or settings (e.g., feedback, reminders etc.)

Social support healthcare personnel
Get feedback & ask questions

Rewards
Points and trophies when reaching goals & target

Metaphors
Motivating metaphors e.g., a garden of flowers: every time a target is reached a flower grow

Self-monitoring
Registrations see connections & possibilities for change

Social support other users
Communication with other users, sharing experiences, competition, cooperation.

Visualization
Presentation of content through animations, graphs, cartoons etc.

Tailoring
Tailoring / adapts based on personal use, e.g., content & tasks based on earlier preferences, time, place.

Feedback
Provides feedback, e.g. on past achievements, rewards and progression in relation on targets & behavior.
