# Supplementary material for: Identifying Persuasive Design Principles and Behavior Change Techniques Supporting End User Values and Needs in eHealth Interventions for Long-Term Weight Loss Maintenance: Qualitative Study
Source: J Med Internet Res. 2020 Nov 30;22(11):e22598. doi: 10.2196/22598 (PMC7735908; doi:10.2196/22598)
Supplement: Multimedia Appendix 3 [file jmir_v22i11e22598_app3.pdf]

Multimedia Appendix 3 Value specification, persuasive system design (PSD) principles and behavior change techniques (BCT) identification, and high-level requirements development for eHealth interventions supporting weight loss maintenance

Key values (WHY)								Quote Prospective End-Users* / Other Key Stakeholders**	User needs / attributes (WHAT)	PSD Principles / BCT Techniques	High-Level Requirements (HOW)	Example Design Features
Personalized care	Feel supported	Positive self-image	Health	Happiness	Motivation	Autonomy	Self-management	*End-user and other key stakeholder example quotes from the individual interviews.*	*Identified end-user needs to maintain weight.*	*Identified PSDs and BCTs to meet end-user values and needs.*	*Illustrate how identified PSDs and BCTs can be combined to meet end-user values and needs.*	*Illustrate how the high-level requirements can be operationalized / implemented in technology design.*
**Value Specification**										**Requirements Development**		
X						X		I would like to choose my own habits, goals and sub-goals to work on, such as reaching a weight target, eating more fruit or walk 6000 steps per day, to keep track of my own progress*	To choose by myself – be in charge	Personalization<br>Goals and planning(outcome)	The system allows the user to choose & adjust own weight goals, and (evidence based) healthy behaviors/habits to maintain weight.	Goal setting and planning of performance of healthy behaviors (e.g., daily walking goal and healthy eating habits) (personal choice). Planning of performance of the behavior should be specified (e.g., context, frequency, duration, intensity).
X								I would like the system to provide relevant feedback and information that fits me*	Personalized content/care	Tailoring<br>Personalization<br>Feedback (on behavior)<br>Shaping knowledge	The system provides knowledge, tailored information and feedback, based on evidence-based knowledge, personal preferences and user data (e.g., related to weight zones for weight control, chosen goals and habits).	Adaptive, tailored feedback (e.g., related to time or performance of behaviors) based on automatic (e.g., sensors) or manual registrations (prompts/cues). The user can access information, mark favorites, set reminders (personal choice).
		X			X			It is really important that the technology motivates and support me to keep up... If the technology would focus on what is going bad, I would feel guilty and stop using it…*	Reinforcing the positive, no punishment for unaccomplished goals/slacking	Praise<br>Rewards<br>Feedback and monitoring	The system positively reinforces accomplishments, presents accomplished goals, rewards, and/or provides coping strategies / exercises (e.g., focus on past success).	Motivating messages and rewards to stay motivated on good and bad days (e.g., after overeating, when weight increase), or related to goals / already accomplished behavior.
	X				X			Knowledge is less important in the maintenance phase, as many know a lot by then. They need a motivator, somebody cheering on the sideline, to be able to continue with the healthy habits to maintain weight**	Positive approach for support and motivation to keep up with good routines and healthy habits	Praise<br>Feedback<br>Tailoring<br>Social role<br>Social support	The system provides positive message and tailored feedback on behavior.	Motivational, positive feedback messages and/or a Coach (virtual/animated).

**Key values (WHY)**								**Quote Prospective End-Users* / Other Key Stakeholders****	**User needs / attributes (WHAT)**	**PSD Principles / BCT Techniques**	**High-Level Requirements (HOW)**	**Example Design Features**
Personalized care	Feel supported	Positive self-image	Health	Happiness	Motivation	Autonomy	Self-management	*End-user and other key stakeholder example quotes from the individual interviews.*	*Identified end-user needs to maintain weight.*	*Identified PSDs and BCTs to meet end-user values and needs.*	*Illustrate how identified PSDs and BCTs can be combined to meet end-user values and needs.*	*Illustrate how the high-level requirements can be operationalized / implemented in technology design.*
						X	X	For good days: a habit tool-box - with habit suggestions for habit formation... And on a bad day or periods: tools to get help in difficult situations**	Support on good and bad days	Suggestion<br>Reduction<br>Rehearsal<br>Repetition and substitution<br>Planning	The system supports establishment of healthy behaviors/habits and supports the user to train on difficult situations/deal with unhealthy behavior and risk situations.	The user can choose between healthy habits related to healthy behaviors to maintain weight (e.g., physical activity, healthy eating, to gradually change behavior/ lifestyle). The system prompt suggestions of healthy snacks before the weekend.
		X				X		I would like to feel I can manage my weight. I don´t want to destroy what I have reached so far*	Be in charge – confidence	Self-belief<br>Goals and planning<br>Shaping knowledge	The system should prompt the users to make a list of what they have achieved so far and why reaching these changes is important.	Self-reflection exercise and feedback to strengthen belief that users are able to maintain weight and continue with healthy behaviors (e.g., prompt the user to analyze barriers or factors influencing the behavior and/or select strategies that facilitate healthy behaviors).
				X	X			Digital tools can help to stay motivated, like real-time connection with a smartwatch to see how much steps I´ve taken, and how much kcal that equals… it must be fun and easy*	Tools to self-monitor to learn/gain insight that stimulate joy and positive feelings	Self-monitoring<br>Tailoring<br>Feedback<br>Praise	The system support joyful, engaging design and features like real time (automatic) monitoring and tailored feedback through visuals, like graphs etc.	Self-monitoring of behavior and positive tailored, adapted feedback related to the user’s performance / behavior.
				X				Healthy diet and good health should be something fun and nice, not something that gives me a bad conscious and demands a lot of energy*	Focus on enjoyableness, realistic goal setting, give experience of mastery	Liking<br>Rewards	The system includes self-selected rewards and enjoyable elements, and/or motivational feedback to stimulate autonomous motivation (e.g., inspirational quotes, challenges, fun facts, rewards, suggestions).	Gamification elements; Earn points and/or get rewards related to self-selected targets / behavior (to maintain and improve yourself).
X				X			X	A technology that gives joy and a sense of meaning or added value will be used**	Useful	Personalization<br>Goals and planning<br>Feedback and monitoring	The system includes enjoyable or surprising elements such as inspirational quotes and tips, praise based on personal preferences and use of the system.	Look and feel of the technology that inspires and engages the users in reaching their targets. The user is able to personalize and to set own preferences related to for example colors, name of animated coach, reminders etc.

Key values (WHY)								Quote Prospective End-Users* / Other Key Stakeholders**	User needs / attributes (WHAT)	PSD Principles / BCT Techniques	High-Level Requirements (HOW)	Example Design Features
Personalized care	Feel supported	Positive self-image	Health	Happiness	Motivation	Autonomy	Self-management	*End-user and other key stakeholder example quotes from the individual interviews.*	*Identified end-user needs to maintain weight.*	*Identified PSDs and BCTs to meet end-user values and needs.*	*Illustrate how identified PSDs and BCTs can be combined to meet end-user values and needs.*	*Illustrate how the high-level requirements can be operationalized / implemented in technology design.*
	X						X	Repetition is the clue and regular confirmation. People need that to stay motivated and not to fall back into old routines. If they fall back, they lose their confidence even more... **	Self-regulation, training on new habits and realistic goal setting	Rehearsal<br>Goals and planning<br>Repetition and substitution<br>Feedback and monitoring	The system support training and rehearsal of new healthy habits and behaviors.	Stimulate/facilitate training/rehearsal of healthy behaviors/habits by supporting self-mentoring of goals, habits and providing motivational feedback on behavior and outcomes.
		X				X		The person needs to feel that they are the expert, their own support and helper**	Be in charge	Self-belief<br>Identity	The system provide exercise e.g., to focus on past success and positive self-image via feedback messages and positive self-talk.	Provide exercise/information to the user (e.g., to reflect on or write down personal strengths and prompt positive self-talk).
					X			It is easier to stay motivated when weight goes down than when it stops. Then you feel that all effort is not rewarded. We try to focus on their health and well-being – on the positive effects. Some still feel it costs more than it benefits to maintain weight**	Receive instant (short term) reinforcement related to values and health outcomes, understand benefits of healthy habits	Natural consequences<br>Comparison of outcomes<br>Covert learning	The system support users in their behavior change process in a motivational way by providing information on health benefits / effects of healthy behaviors.	Imaginary rewards and information about pros and cons of healthy vs. unhealthy behaviors.
							X	I would like to understand which habits and behaviors that works and does not work, to be able to stay healthy and maintain my current weight*	Comprehension of own process (behavior change)	Feedback<br>Simulation<br>Shaping knowledge<br>Goals and planning	The system should provide means for understanding the link between cause and effect with regard to behaviors (healthy diet & physical activity) and weight (maintenance). (e.g., Overview of the connection between behavior in relation to weight, long-term storage of data).	Self-monitoring and visualization of data related to outcome and behavior (e.g. graph and/or personal overview based on registered information/data). A personal overview (e.g., over weight and targets/behavior), where the user can access specific day/week (past/present).
							X	How to prevent overeating, and avoid unhealthy temptations… both tools, learning from others and know how the body works can help*	Strategies to self-regulate (emotional eating & temptations)	Social support (PSD: e.g., social learning, social comparison, social facilitation, cooperation)<br>Social comparison (BCT)<br>Goals and planning<br>Regulations<br>Antecedents	The system advice to introduce a social stimulus linked to a certain behavior, provide strategies/exercises (e.g., to make "if-then plans"/coping planning for weight regain and relapse prevention, problem solving) and/or	Strategies / exercises are offered to identify and train on situations up front (e.g., cognitive diamond; awareness of relations between thoughts, behavior, feeling, body sensation).

Key values (WHY)								Quote Prospective End-Users* / Other Key Stakeholders**	User needs / attributes (WHAT)	PSD Principles / BCT Techniques	High-Level Requirements (HOW)	Example Design Features
Personalized care	Feel supported	Positive self-image	Health	Happiness	Motivation	Autonomy	Self-management	*End-user and other key stakeholder example quotes from the individual interviews.*	*Identified end-user needs to maintain weight.*	*Identified PSDs and BCTs to meet end-user values and needs.*	*Illustrate how identified PSDs and BCTs can be combined to meet end-user values and needs.*	*Illustrate how the high-level requirements can be operationalized / implemented in technology design.*
											social peer support (e.g., user stories / groups).	
							X	Weight maintenance is all about planning. I would like to have different plans for different situations, a plan A for «normal days and weeks» and a plan B: when «crisis occurs», cake at work or holidays, to be able to withstand the constant feeling of hunger and temptation situations*	Having a good plan gives peace of mind. Help and/or coping strategies in dealing with tempting / challenging situations	Personalization<br>Goals and planning<br>Associations<br>Antecedents	The system should support the users in problem solving, to identify barriers related to goals and plans (e.g., practical, social and emotional barriers), and include a personalized planning tool for different situations (e.g., risk situations, crisis, holiday).	Support the user to make personal plans for different situations in order to cope/master (e.g., planning tool with calendar function). The plan should be easy to access (just-in-time) when needed (e.g., coping plans for risk situations).
							X	Many have a lot of knowledge about what to do, but they fail to actually do it in practice**	Self-regulation, motivation & support to establish healthy habits	Repetition and substitution<br>Goal and planning<br>Associations<br>Antecedents	The system support training and rehearsal of new healthy habits and behaviors, through small steps, easy to perform tasks.	Prompt the user to practice or rehearsal/repetition of behavior and self-management strategies in relation to context or time.
	X				X			Motivation, tips and advice - back-up and support is really needed, since I don´t always get that from my family... It demotivates when my surroundings don´t believe I can make it. It motivates me when they say – you can do it! *	Confirmation and tailored (social) support when needed	Tailoring<br>Suggestions<br>Social role<br>Social support	The system includes positive, joyful support and social features; social support (human or virtual), and/or cooperation / connection with other users; group / peer interaction.	Social support: Virtual / animated coach, chat, personal pages with practical tips, virtual groups, etc.
					X			What is "motivating" about weight maintenance. I need to set some new goals and milestones, maybe a training goal, to keep up*	Be driven to change, find motivation of the user, goals related to values, purpose and preferences	Personalization<br>Goals and planning	The system supports the users in eliciting purpose of behavior change, realistic goal setting.	Personalized goals & plans (e.g., target behavior), and prompt planning the performance of healthy behavior (e.g., when to do which physical activity)
			X					We try to focus on their health and well-being – on the positive effects – some still feel the costs are more than it benefits**	Understand benefits of healthy habits/behaviors	Feedback and monitoring<br>Natural consequences	The system allows the users to learn about healthy habits / behaviors and related health effects.	Provide information and knowledge about health consequences (written or visual) of performing healthy habits / behavior.

**Key values (WHY)**								**Quote Prospective End-Users* / Other Key Stakeholders****	**User needs / attributes (WHAT)**	**PSD Principles / BCT Techniques**	**High-Level Requirements (HOW)**	**Example Design Features**
Personalized care	Feel supported	Positive self-image	Health	Happiness	Motivation	Autonomy	Self-management	*End-user and other key stakeholder example quotes from the individual interviews.*	*Identified end-user needs to maintain weight.*	*Identified PSDs and BCTs to meet end-user values and needs.*	*Illustrate how identified PSDs and BCTs can be combined to meet end-user values and needs.*	*Illustrate how the high-level requirements can be operationalized / implemented in technology design.*
			X		X			The benefits of daily activities like walks should be emphasized. Small changes can give large health effects, to understand these mechanisms and behavioral patterns motivate. It takes much more time and energy than earlier to plan and keep up with the new, healthy habits*	Understand benefits of healthy habits/behaviors, creating and sticking to habits	Feedback and monitoring<br>Natural consequences	The system allows the users to monitor healthy habits / behaviors and provides information about of habit formation and benefits of small steps/behavior change.	Provide knowledge about health effects of healthy habits (e.g., walk/bike to work instead of driving), and monitoring of healthy habits and behaviors in an engaging way.
