# Supplementary figures and images for: Identifying Persuasive Design Principles and Behavior Change Techniques Supporting End User Values and Needs in eHealth Interventions for Long-Term Weight Loss Maintenance: Qualitative Study
Source: J Med Internet Res. 2020 Nov 30;22(11):e22598. doi: 10.2196/22598 (PMC7735908; doi:10.2196/22598)

## Multimedia Appendix 4

Infographics to visualize the demographics of included end users.

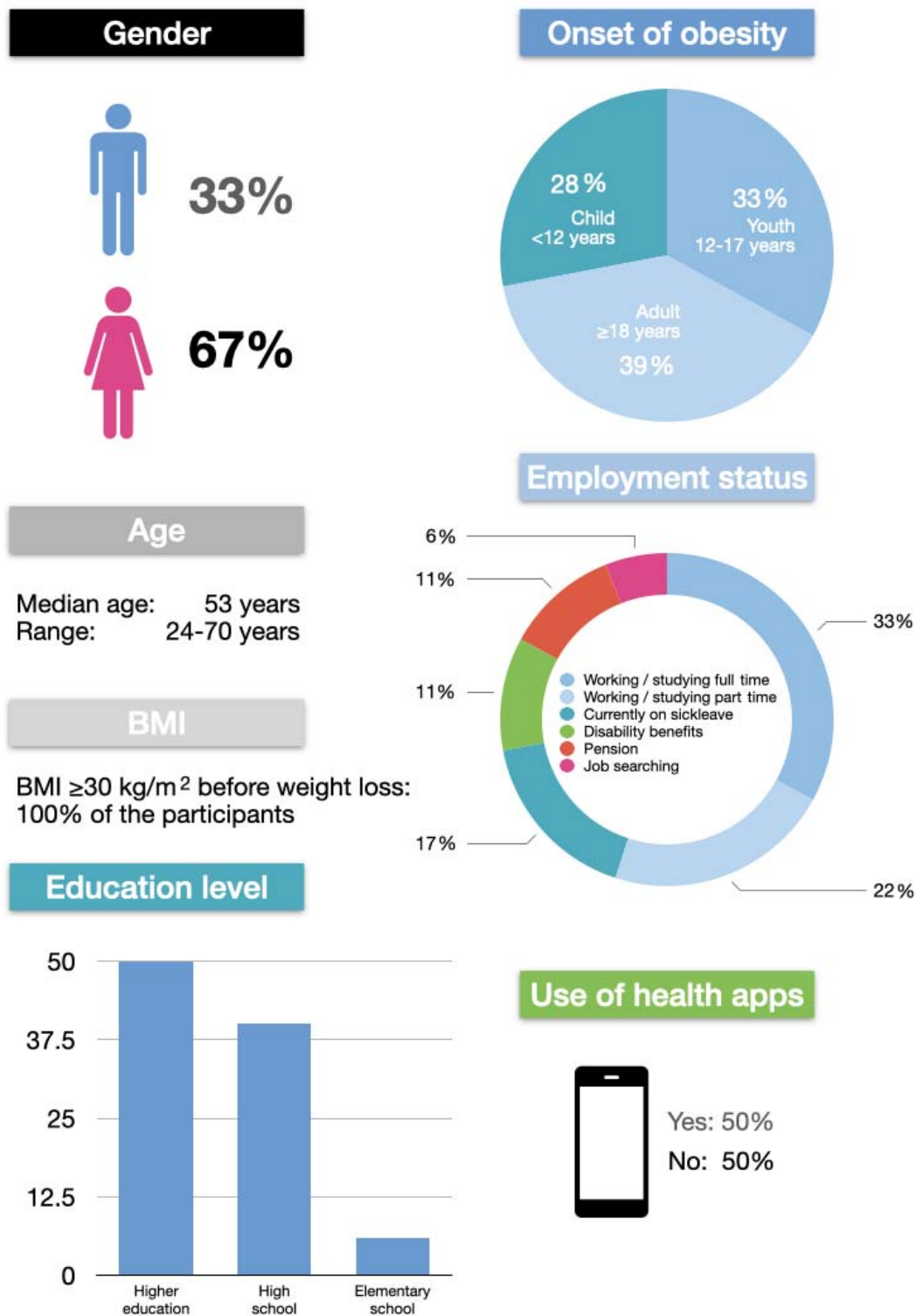

Supplement: Multimedia Appendix 4 [file jmir_v22i11e22598_app4.pdf]
